# Immunological Mechanisms and Machine Learning Applications in Post-COVID-19 Syndrome: A Narrative Review

**DOI:** 10.3390/microorganisms14061313

**Published:** 2026-06-11

**Authors:** Leonid P. Churilov, Anna Starshinova, Igor Kudryavtsev, Artem Rubinstein, Olesya Koroteeva, Anastasia Kulpina, Varvara A. Ryabkova, Adilya Sabirova, Polina Sobolevskaia, Tamara Fedotkina, Dmitry Kudlay

**Affiliations:** 1 Institute of Medicine, Department of Pathology, St. Petersburg State University, 199034 St. Petersburg, Russiavarvara-ryabkova@yandex.ru (V.A.R.);; 2 Faculty of Mathematics and Computer Science, St. Petersburg State University, 199034 St. Petersburg, Russia; 3 Almazov National Medical Research Centre, 197341 St. Petersburg, Russia; 4 Medical Department, Bashkir State Medical University, 450008 Ufa, Russia; 5 Institution of Experimental Medicine, Department of Immunology, 197376 St. Petersburg, Russia; 6 Medical Department, I.P. Pavlov 1st Saint Petersburg State Medical University, 197022 St. Petersburg, Russia; 7 Sechenov Institute of Evolutionary Physiology and Biochemistry of the Russian Academy of Sciences, 194223 St. Petersburg, Russia; 8 Department of Pharmacology, Institute of Pharmacy, I.M. Sechenov First Moscow State Medical University, 119435 Moscow, Russia; 9 Institute of Immunology FMBA of Russia, 115478 Moscow, Russia; 10 Faculty of Bioengineering and Bioinformatics, Lomonosov Moscow State University, 119991 Moscow, Russia

**Keywords:** autoimmunity, artificial intelligence, disease prediction, long COVID, lymphocyte subsets, machine learning, systematic review, PRISMA, neuroendocrine regulation

## Abstract

Post-COVID-19 syndrome (PCS), also referred to as post-acute sequelae of SARS-CoV-2 infection (PASC), represents a heterogeneous set of persistent clinical manifestations developing after acute infection. These conditions are associated with immune dysregulation, autonomic imbalance, impaired thymic function, and possible viral persistence. Objective: This study aims to systematically synthesise current evidence on the immunopathogenesis of PCS and to critically evaluate the application of artificial intelligence (AI) and machine learning (ML) approaches for its prediction and clinical stratification. Methods: A PRISMA 2020–informed systematic review was conducted using PubMed/MEDLINE, Scopus, Web of Science, elibrary.ru and Embase databases (January 2020–December 2025). Studies addressing immunopathological mechanisms and AI/ML applications in PCS were selected based on predefined eligibility criteria. Risk of bias in prediction studies was assessed using the PROBAST tool. Due to heterogeneity, a structured qualitative synthesis was performed. Current evidence indicates that PCS may result from sustained systemic inflammation, cytokine dysregulation, autoimmunity, and delayed restoration of T-cell homeostasis, including reduced thymic output of naïve T lymphocytes. Persistent thymic dysfunction may contribute to prolonged immune imbalance, increased susceptibility to secondary infections, and reactivation of latent viruses. AI/ML approaches—including gradient boosting, ensemble learning, deep neural networks, and natural language processing—have demonstrated promising performance across multimodal datasets. However, significant limitations were identified, including small sample sizes, overfitting, lack of external validation, and heterogeneity in outcome definitions. Conclusions: The integration of immunopathological insights with data-driven modelling highlights the potential of combined approaches for improving PCS risk stratification. However, current AI models remain insufficiently validated for clinical implementation. Future research should prioritise methodological standardisation, external validation, and incorporation of mechanistically informed biomarkers.

## 1. Introduction

Post-COVID-19 syndrome (PCS) has emerged as a major medical and societal challenge in the post-pandemic period. Symptoms persisting or emerging ≥ 3 months after SARS-CoV-2 infection occur in approximately 10–20% of those infected [1,2,3].

The prevalence of PASC (Post-acute Sequelae of COVID-19) is associated with prolonged disability, reduced quality of life, and an increasing burden on healthcare systems [4,5,6].

Estimates of PCS prevalence vary across studies, reflecting differences in populations and definitions. In severe COVID-19, the risk of PCS is significantly higher. In severe COVID-19, the risk of PCS is significantly higher [7]. The pathogenesis of PCS is multifactorial and includes viral persistence, chronic immune activation, antigenic mimicry, autoimmune reactions, cytokine imbalance, endothelial dysfunction, and microthrombosis [8,9,10]. These mechanisms may interact in a patient-specific manner, forming heterogeneous clinical and pathogenetic phenotypes, from cardiorespiratory to neurocognitive and metabolic [11]. Emerging evidence also suggests involvement of neuroendocrine and autonomic regulatory systems, although their precise role remains to be clarified [12]. Bioinformatic methods were used to analyze the manifestations of antigenic mimicry of coronavirus antigens and marker autoantigens of hypophysitis, adrenalitis, autoimmune thyropathies, small fiber neuropathies, arteritis, as well as receptors of the autonomic nervous system (ANS), gonadal autoantigens and proteins involved in atherogenesis [13,14,15,16,17]. A cell-mediated immune mechanism of damage to the brain and parenchymal cells of neuroendocrine organs was demonstrated, documented by pathological cellular infiltration predominantly by CD4^+^ and CD8^+^ T lymphocytes and CD20^+^ B cells [12,18,19]. The characteristics of the autonomic nervous system (ANS) lesion have been clarified, and signs of polyneuropathy of the smallest fiber diameter, with sensory and autonomic dysfunction, have been identified. Polyclonal activation of humoral autoimmunity has been noted [20,21].

Phenotypes of ANS with signs of adrenal insufficiency (in the presence of autoantibodies to adrenocorticocytes) and with constitutional features of connective tissue and blood, as well as microbiota, have been described [22,23,24]. With the growing volume of clinical and laboratory data on the pathogenesis of ACL [25], it has been shown that traditional statistical models, when generalized, have low predictive power due to high interindividual variability and incomplete information in electronic medical records [26].

Machine learning (ML) methods represent a promising approach for integrating multidimensional clinical, laboratory, and imaging data [27]. ML models enable the identification of hidden nonlinear relationships between biomarkers, immunobiochemical profiles, and clinical outcomes, providing a personalized prognosis and the basis for pathogenetically based patient stratification.

This study aims to systematically synthesise current evidence on the immunopatho-genesis of PCS and to critically evaluate the application of artificial intelligence (AI) and machine learning (ML) approaches for its prediction and clinical stratification.

## 2. Materials and Methods

This review was conducted in accordance with the Preferred Reporting Items for Systematic Reviews and Meta-Analyses with the use PRISMA 2020 [28] guidelines. A predefined protocol was developed to ensure transparency in study identification, selection, and synthesis.

A comprehensive search was performed in PubMed/MEDLINE, Embase, Scopus, Web of Science, and eLibrary databases from 1 January 2020, to 31 December 2025. Database-specific search strings were adapted according to indexing systems and controlled vocabularies. For PubMed, the principal search strategy was: (“Long COVID” OR “Post-COVID Syndrome” OR “Post-Acute Sequelae of SARS-CoV-2 Infection” OR PASC) AND (“immune dysregulation” OR cytokine OR autoimmunity OR thymus OR “T-cell homeostasis”) AND (“artificial intelligence” OR “machine learning” OR “deep learning” OR XGBoost OR NLP OR “predictive modelling”).

### 2.1. Eligibility Criteria

Studies were considered eligible if they met the following criteria:

(i) Original research articles, systematic reviews, or high-quality meta-analyses;
(ii) Studies involving adult or mixed populations with post-COVID-19 syndrome (PCS) or post-acute sequelae of SARS-CoV-2 infection (PASC);
(iii) Investigations addressing immunopathological mechanisms (e.g., cytokine dysregulation, autoimmunity, thymic function, T-cell homeostasis) and/or applications of artificial intelligence (AI) and machine learning (ML) in diagnosis, prediction, or risk stratification;
(iv) Publications in peer-reviewed journals;
(v) Articles published in English and other languages.

### 2.2. Exclusion Criteria Included

(i) Case reports or small case series (*n* < 10);
(ii) Conference abstracts without full text;
(iii) Non-peer-reviewed sources (unless used for contextual discussion);
(iv) Studies lacking clear methodological description;
(v) Duplicate publications.

A comprehensive literature search was performed across the following electronic databases: PubMed/MEDLINE, Scopus, Web of Science, elibrary.ru and Embase. The search covered the period from January 2020 to December 2025.

Search terms included combinations of controlled vocabulary (e.g., MeSH terms) and free-text keywords related to “post-COVID syndrome”, “long COVID”, “PASC”, “immune dysregulation”, “thymus”, “T-cell homeostasis”, “autoimmunity”, “cytokines”, “machine learning”, “artificial intelligence”, “deep learning”, “NLP”, “XGBoost”, and “predictive modelling”.

### 2.3. Study Selection

All retrieved records were imported into a reference management system, and duplicates were removed. Two independent reviewers screened titles and abstracts for relevance. Full-text articles were subsequently assessed for eligibility based on predefined inclusion and exclusion criteria. Discrepancies between reviewers were resolved through discussion or consultation with a third reviewer.

**Figure 1:**
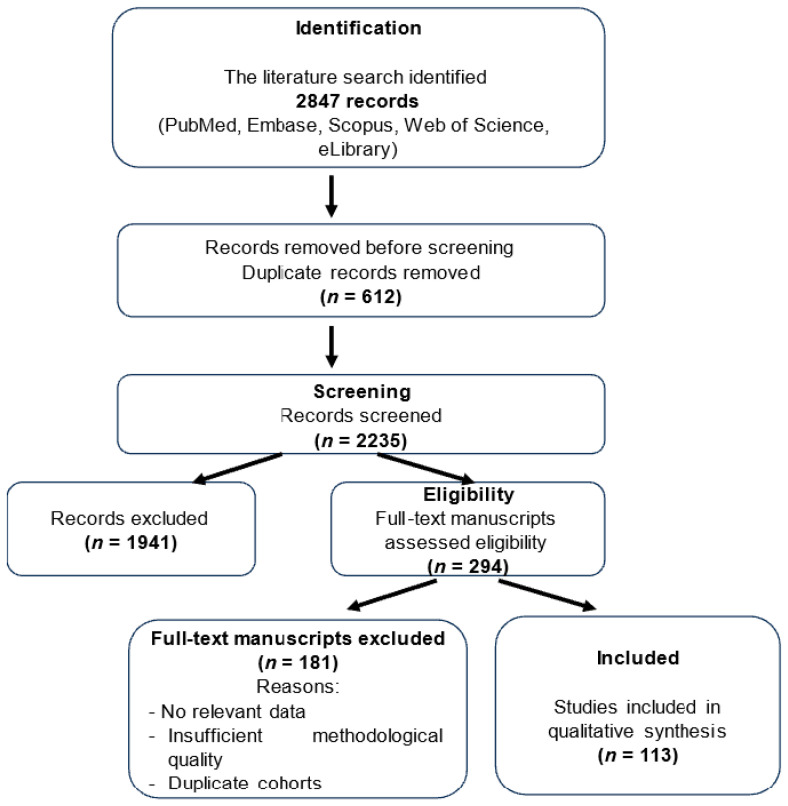
A detailed study selection process is presented in the PRISMA 2020 flow diagram.

The literature search identified 2847 records. After removal of 612 duplicates, 2235 records underwent title and abstract screening. Of these, 1941 records were excluded because they did not meet eligibility criteria. The full texts of 294 articles were assessed, resulting in the exclusion of 181 studies due to insufficient methodological quality, absence of relevant immunological or AI/ML data, or duplication of reported cohorts. Ultimately, 113 studies were included in the qualitative synthesis. A detailed study selection process is presented in the PRISMA 2020 flow diagram (Figure 1).

### 2.4. Risk of Bias Assessment

Prediction studies were evaluated using the Prediction model Risk Of Bias ASsessment Tool (PROBAST). Four domains were assessed: participants, predictors, outcomes, and analysis. Each study was independently assessed by two reviewers. Disagreements were resolved by consensus. Studies were classified as having low, moderate, or high risk of bias according to PROBAST recommendations.

Among the included AI/ML studies, 21% were classified as low risk of bias, 46% as moderate risk, and 33% as high risk. The highest frequency of bias was observed in the analysis domain, primarily due to insufficient external validation, inadequate reporting of calibration, and potential overfitting.

### 2.5. Sensitivity and Limitations

Potential sources of bias include heterogeneity in PCS definitions, variability in follow-up duration, and differences in AI model validation practices. These factors were considered when interpreting the findings.

## 3. Immunological Mechanisms Relevant to Post-COVID-19 Syndrome Prediction

The number of patients with post-COVID-19 syndrome (PCS) remains substantial in the post-pandemic period. Long-term consequences of SARS-CoV-2 infection contribute to the burden of chronic disease. The consequences of prior coronavirus infection complicate the course of comorbid chronic cardiovascular, pulmonary, and renal diseases in a vast number of patients. For example, coronavirus infection has been recognised as a novel atherogenic factor [29,30,31,32]. These factors highlight the ongoing clinical and societal importance of PCS.

The use of machine learning models—including interpretable ones (such as logistic regression and decision trees), ensemble learning methods (e.g., optimised XGBoost, stacking), neural networks (FNN, RNN, CNN), and contemporary explainable artificial intelligence (AI) technologies (such as SHAP)—not only enhances predictive accuracy but also improves our understanding of PCS pathogenesis.

PCS reflects complex interactions between immune, inflammatory, and neuroendocrine systems operating across multiple biological levels. These interactions cannot be explained by a single pathway but arise from coordinated and sometimes conflicting regulatory processes. It would be an oversimplification to consider the response to infection or tissue injury merely as inflammation [28,33,34]. Several typical compensatory and adaptive processes of various levels are activated both in parallel and sequentially, as cells and the organism as a whole possess stereotypical programmed responses that are automatically triggered by disease or injury. The organism’s response as a system to damage is complex and multi-level but remains within the framework of programmed reactivity stereotypes. These stereotypes include both ancient ones inherent to cellular responses and more recent ones that evolved in higher animals. The former depend on short-range bioregulators—juxta- and paracrine mediators—produced by the cells themselves (known in pathophysiology as inflammatory mediators or, more broadly, as autacoids). A specific subset of these bioregulators is represented by cytokines. The latter depend on long-range systemic regulators produced by the neuroendocrine system, such as hormones and neurotransmitters [9,35].

Any significant injury—and especially acute COVID-19 infection—simultaneously triggers programmed cell–tissue (local) and systemic–integrative responses. When these programmes do not conflict, both protective pathways remain within the bounds of moderate pathogenicity and sufficient defensive efficacy [36,37]. However, under certain conditions, the parallel effects of these bioregulators may lead to conflicts that compromise the optimal execution of protective programmes, particularly in the context of extensive injury, severe acute disease, or limited compensatory resources. This results in mutual attenuation of their protective potential and an increased cost of adaptation, eventually leading to the predominance of secondary pathogenicity of the protective mechanisms themselves. Such a typical conflict between local immune-dependent and systemic neuroendocrine mechanisms forms the basis for chronic as well as acute health disturbances [38].

In general pathology and in specific clinical and pathophysiological studies, the vast majority of works focus on individual pathological processes as elements of disease. Considerably less attention has been paid to the conflicting or synergistic interactions between different pathological processes and to the study of how these pathocybernetic interactions influence resource allocation, the cost of adaptation, and the pathogenic consequences of compensatory processes. Yet disease as a whole represents a disturbance of informational processes rather than merely material and energetic ones. This principle, known since the works of F.E. Yates as the information–substance dualism [39], remains central to systems biology and systems pathobiology, into which modern pathophysiology is now transforming. Such pathoinformational conflicts, by disorganising the immuno–neuro–endocrine apparatus, may underlie both acute complications of disease and chronic or prolonged health disturbances that persist after apparent recovery from acute conditions. This mechanism, in particular, accounts for the high pathogenicity of the cytokine storm in COVID-19, which represents a specific case of infectious circulatory shock triggered by excessive systemic action of pro-inflammatory mediators disrupting the regulation of vital systemic functions [23]. A classic example of chronic pathology resulting from sustained conflict among multilevel protective programmes is the metabolic syndrome, where excessive systemic activity of inflammatory mediators impairs the efficiency of systemic insulin regulation [40]. According to the authors’ hypothesis [1,41,42,43], such conflicts play a significant pathogenic role in post-COVID-19 syndrome of various phenotypes.

## 4. Main Pathogenetic Mechanisms of Post-COVID-19 Syndrome

Studies investigating the pathogenesis of post-COVID-19 syndrome (PCS) have identified several key mechanisms. Many of these studies suggest that PCS is associated with excessive systemic activity of inflammatory mediators, immune dysregulation, autoimmunity, and abnormalities of cytokine regulation. Other research indicates that prolonged COVID may be related to the persistence of SARS-CoV-2 reservoirs within tissues and to viral reactivation [44].

It is known that SARS-CoV-2 can enter cells expressing angiotensin-converting enzyme 2 (ACE2), but not those expressing other coronavirus receptors, such as aminopeptidase N or dipeptidyl peptidase 4 (DPP4). Since ACE2 serves as the cellular receptor for SARS-CoV-2, many organs and tissues that express this enzyme—including the respiratory system, the immune system, and the neuroendocrine apparatus—are directly affected by COVID-19 [45,46,47].

A decline in thymic function and, consequently, a reduction in the output of naïve T cells may exacerbate lymphopenia in patients with acute COVID-19 and prolong the time required for restoration of circulating T-cell counts and function after recovery. In acute SARS-CoV-2 infection, low or absent levels of T-cell receptor excision circles (TREC) in the circulation have been identified as indicators of an unfavourable disease outcome, as demonstrated in a series of independent studies. For example, a study reported that patients with COVID-19 who developed acute respiratory distress syndrome (ARDS) exhibited significantly lower concentrations of TREC and kappa-deleting recombination excision circles (KREC) than patients without ARDS [48]. Low TREC levels in the acute phase of COVID-19 are considered a predictor of poor prognosis, whereas elevated TREC and KREC concentrations are associated with favourable outcomes [49].

More recently, it has been demonstrated that the human thymus is a direct target of SARS-CoV-2 infection, and its function is markedly impaired following viral entry. Therefore, monitoring thymic activity may represent an important prognostic marker for assessing disease severity and progression [50]. Our own findings regarding T-lymphocyte activity in psychoneuroimmunoendocrine pathology also indicate that thymic dysfunction persists even after apparent recovery from the acute phase of illness, underscoring the importance of evaluating thymic functional activity during convalescence [51].

Furthermore, delayed or incomplete restoration of thymic function may contribute to the development of secondary infections that exacerbate disease severity during both the acute and post-acute phases [52] and promote the persistence of symptoms related, for example, to herpesvirus reactivation during recovery [53]. Such pronounced disturbances in T-cell pool formation in the post-COVID-19 period may influence the course of underlying diseases—in our cohort, notably chronic pulmonary sarcoidosis [54]. Moreover, as reported by our group and others, one of the most significant sequelae of coronavirus infection is the development of granulomatous inflammation. A broad spectrum of clinical cases has been documented in which granulomas formed in patients after COVID-19. The key bioregulator promoting granulomatous processes, tumour necrosis factor alpha (TNF-α), is abundantly produced and exerts both local and systemic effects during SARS-CoV-2 infection and its chronic pulmonary complications [55].

### 4.1. B Cells and Follicular T Helpers in Post-COVID-19

Hyperactivation of B lymphocytes and disturbances in their subpopulation composition have been observed after recovery from acute COVID-19, which may represent a component of post-COVID-19 syndrome and contribute to an increased risk of autoimmune reactions among convalescents. Both the relative and absolute numbers of activated B cells remain elevated after recovery [56]. These cells exhibit an “activated” phenotype (CD19^+^CD80^+^/CD86^+^). The same authors also reported a high proportion of plasma cells and circulating plasmablasts in peripheral blood post-recovery, characterised by high surface expression of PD-1 [56,57]. Castleman et al. further demonstrated not only increased CD86 expression on B cells in recovered patients, but also elevated levels of the activation marker CD69 on CD19^+^ lymphocytes [58,59,60]. Elevated numbers of circulating memory B cells have also been described in convalescents [61]. However, these cells may acquire autoreactive properties following infection. For example, Vijayakumar et al. found that patients who had experienced acute respiratory distress syndrome (ARDS) as part of the post-COVID-19 respiratory syndrome displayed elevated numbers of IgD^−^CD27^+^ memory B cells in the airways, correlating with respiratory abnormalities on computed tomography [62,63,64].

These findings indicate that SARS-CoV-2 infection disrupts B-cell function and alters the expression of major B-cell markers. For instance, one year after severe COVID-19, patients exhibit reduced CD19 expression on B cells, which may affect B-cell activation upon antigen recognition. Moreover, in patients recovering from severe disease, loss of CD21—a molecule involved in transmitting activation signals from the B-cell receptor—has been observed on the B-cell surface, a feature typical of activated autoreactive B cells [58,65,66,67]. The same investigators also reported reduced expression of inhibitory molecules CD22 and CD72 on B cells. Stimulation of the B-cell receptor in such cells led to hyperactivation of downstream effectors, including pSYK, pBLNK and pPLCγ2 [58,68].

The primary function of B cells is antibody production, forming a cornerstone of humoral adaptive immunity in both infectious and autoimmune processes. Increasing evidence suggests the activation of autoreactive B-cell clones in patients with acute and convalescent COVID-19 [69] (Table 1).

Collectively, autoantibodies in PCS appear to converge on three principal axes: (i) autonomic receptor dysfunction, (ii) endothelial and coagulation disturbances, and (iii) breakdown of immune tolerance.

Rather than isolated findings, these autoantibodies likely represent overlapping immunological endotypes that may explain the heterogeneity of PCS clinical phenotypes.

Moreover, convalescent individuals frequently exhibit autoantibodies targeting various cytokines (IFN-α, IFN-ω, IFN-γ, IL-1β, IL-6, IL-10, IL-17, IL-21, GM-CSF) and chemokines (CCL2, CXCL1, CXCL7, CXCL13, CXCL16) in peripheral blood [70]. Some researchers have also detected elevated titres of autoantibodies against chromatin, cardiolipin, and the Smith antigen [58], commonly associated with systemic lupus erythematosus (SLE) [71,72].

During acute COVID-19, the presence of autoantibodies against cytokines has also been confirmed. Chang et al. (2021) demonstrated that approximately 50% of 147 COVID-19 patients developed new-onset autoantibodies [73]. In another study, among 987 patients with acute SARS-CoV-2 infection, 101 were found to carry circulating neutralising antibodies against interferons—including IFN-ω (13 patients), multiple IFN-α subtypes (36 patients), and both IFN-α and IFN-ω (52 patients) [74,75]. The emergence of antibodies against various IFNs has likewise been observed in classical autoimmune disorders such as SLE (Price et al.) and systemic sclerosis [76]. Similar findings were reported by Wang et al. (2021), who demonstrated elevated levels of autoantibodies targeting immunomodulatory factors—including cytokines, chemokines, complement components, and cell-surface proteins—in COVID-19 patients [77,78]. According to the authors, these autoantibodies impair immune signalling, disrupt intercellular communication, and alter immune-cell composition in circulation, thereby exacerbating the severity of SARS-CoV-2 infection.

Interestingly, Woodruff et al. observed that antibodies against Sm/RNP, Ro, La, and dsDNA were rarely detected in patients with severe COVID-19, whereas antinuclear antibodies (ANA) were found in nearly 40% of such cases. Approximately 40% of patients also exhibited antibodies against carbamylated protein (anti-CarP), implicated in connective tissue destruction in SLE and rheumatoid arthritis [79]. Other studies reported ANA elevation in most patients with ARDS [80], suggesting shared mechanisms of lung injury between SARS-CoV-2 infection and autoimmune flare-ups, including SLE and rheumatoid arthritis [81]. Several independent groups confirmed increased titres of ANA and antineutrophil cytoplasmic antibodies (ANCA) in convalescent patients [82], with ANA positivity potentially linked to post-COVID-19 manifestations such as alopecia [80].

Another example of post-COVID-19 autoimmunity is the appearance of rheumatoid factor, whose elevated serum concentration during the acute phase correlates with severe or critical disease [83]. Autoantibodies targeting platelets have also been described, potentially contributing to disease severity and chronic complications [84,85]. Furthermore, in the results of some studies researches were reported elevated titres of anti-PM-Scl and anti-Scl-70 antibodies in patients who developed pulmonary fibrosis, raising concerns about the long-term sequelae of severe COVID-19 [86,87,88]. Increased levels of antiphospholipid antibodies—anticardiolipin, anti-β_2_-glycoprotein I, and anti-phosphatidylserine/prothrombin—have also been observed during the acute phase [86,89,90].

The development of an effective adaptive humoral response requires interaction between follicular T helper (Tfh) cells and B lymphocytes at the boundary between T- and B-dependent zones of lymphoid follicles [91,92]. Tfh cells play a critical role in B-cell maturation and differentiation within germinal centres and in affinity maturation processes occurring in peripheral lymphoid organs (Tangye et al.). These cells regulate antibody class switching, initiate somatic hypermutations, and mediate the selection of high-affinity B-cell clones that subsequently differentiate into plasma cells and memory B cells. Thus, this subset of CD4^+^ T cells is essential for the production of high-affinity antibodies against pathogens and, under pathological conditions, for autoantibody generation. Identification of CD4^+^CXCR5^+^ T cells is therefore a key parameter in assessing adaptive humoral immune competence in infectious and autoimmune diseases [93,94].

The population of circulating Tfh cells is highly heterogeneous. Based on CXCR3 and CCR6 co-expression, four principal subsets can be distinguished: CXCR3^+^CCR6^−^ Tfh1, CXCR3^−^CCR6^−^ Tfh2, CXCR3^−^CCR6^+^ Tfh17, and CXCR3^+^CCR6^+^ Tfh17.1. These subsets resemble Th1, Th2, “classical” Th17, and “proinflammatory” Th17.1 cells, respectively, in their functions and phenotypes. Dysfunction of Tfh cells and imbalances between these subsets are closely linked to aberrant regulation of humoral immunity. For example, altered proportions of Tfh1 (inhibitory) versus Tfh2/Tfh17 (stimulatory) cells have been reported in rheumatoid arthritis, SLE, Sjögren’s syndrome, multiple sclerosis, and type 1 diabetes [21,61,71,95,96].

During acute COVID-19, Tfh cells determine the efficiency of B-cell responses to viral infection. Through Tfh-mediated stimulation, B lymphocytes undergo refined differentiation, giving rise to long-lived memory B cells and plasma cells producing high-affinity antibodies [97]. Levels of SARS-CoV-2–specific circulating Tfh (cTfh) cells in recovering patients correlate with titres of neutralising antibodies, underscoring their central role in maintaining protective immunity [98]. However, SARS-CoV-2 infection can disrupt Tfh differentiation, impairing humoral responses [99]. Kaneko et al. demonstrated germinal centre atrophy in lymph nodes at autopsy—corresponding to B-cell zones—likely resulting from cytokine-storm–induced TNF-α–mediated inhibition of Bcl-6^+^ Tfh differentiation [100]. This may also relate to a heightened local Th1 response, as Th1/Tfh1 cells suppress follicular T helper differentiation and antibody production [100,101].

Even CD4^+^CXCR5^+^ Tfh cells lacking Bcl-6 can interact with naïve B cells outside follicles, driving extrafollicular proliferation. Thus, during acute SARS-CoV-2 infection, Tfh cells exhibit enhanced functional and proliferative activity, yet differentiation defects negatively affect B-cell–mediated immunity. Moreover, the balance between inhibitory Tfh1 and stimulatory Tfh2/Tfh17 populations is disrupted, with reduced Tfh1 and increased Tfh17 frequencies in peripheral blood [102,103,104].

Similar alterations in circulating Tfh subsets are seen in convalescent individuals. Among recovered patients, higher Tfh1 frequencies correlate with severe acute infection and neutralising antibody titres [105]. Other studies report elevated Tfh1 and Tfh2 with reduced Tfh17 levels [106]. Convalescents display an increased proportion of effector-memory CCR7^lowPD-1^+^ Tfh-em cells and a reduced fraction of central-memory CCR7^highPD-1^+^ Tfh-cm cells compared with healthy controls [106]. In the post-COVID-19 period, several studies have noted persistent Tfh hyperactivity in convalescents [106,107], potentially contributing to autoimmune or allergic manifestations within post-COVID-19 syndrome. The activity of these cells also correlates with the severity of prior SARS-CoV-2 infection: concentrations of Tfh-em cells driving class-switched IgG antibody responses remain elevated long after severe disease [106]. Our preliminary findings show persistently elevated Tfh2, Tfh17, and double-positive Tfh frequencies in convalescents compared with healthy donors, accompanied by reduced CXCR3^+^CCR6^−^ Tfh1 and increased CXCR3^−^CCR6^+^ Tfh17 proportions. This pattern may facilitate autoimmune processes through impaired immune tolerance mechanisms, frequently observed in post-COVID-19 syndrome [104].

### 4.2. Regulatory T Lymphocytes

The principal target cells for regulatory T lymphocytes (Tregs) include immunocompetent cells of both the innate immune system (tissue macrophages, antigen-presenting cells, natural killer cells) and the adaptive immune system (effector cytotoxic T lymphocytes, T helpers, and B lymphocytes). Tregs exert their functions through multiple mechanisms, which are traditionally divided into “non-contact” mechanisms—mediated by the action of soluble molecules secreted by Tregs and diffusing through tissue fluids—and “contact-dependent” mechanisms, mediated by direct receptor–ligand interactions between Tregs and target cells [108].

At present, several classifications of Tregs have been proposed; however, the most informative approach appears to be the division into Th1-like, Th2-like, and Th17-like Tregs. Th1-like Tregs are characterised by the presence of the transcription factor T-bet, CXCR3 expression on the membrane, and production of IFN-γ. Th2-like Tregs express GATA3 and secrete Th2 cytokines, including IL-4, IL-5, and IL-13. Th17-like Tregs are IL-17A–secreting, RORγt-positive lymphocytes that also express CCR6 on their surface [109,110] Among these subsets, CXCR3^+^ Th1-like Tregs are considered the most potent anti-inflammatory cells, as they produce the highest amount of IL-10 in response to in vitro stimulation compared to other Treg types. Furthermore, CXCR3^+^ Th1-like Tregs express the transcription factor T-bet (TBX21), which is typical of Th1-like subsets, and exhibit elevated expression of CD73 and TGF-β1 relative to other Treg subpopulations [89]. These T-bet^+^ Tregs also display high levels of TIGIT expression, which is associated with increased IL-10 production and the ability to drive dendritic cells towards a tolerogenic phenotype [91,111].

During infectious processes, CXCR3^+^ Th1-like Tregs can migrate to sites of pathogen entry and suppress both innate and adaptive immune responses aimed at pathogen elimination. This “pathogenic” role of Tregs is particularly relevant in type 1 inflammatory responses targeting intracellular pathogens, as demonstrated in viral infections, including COVID-19.

In COVID-19, increased circulating levels of Tregs have been associated with severe disease and poor clinical outcomes [112]. Conversely, other studies have shown that Treg numbers progressively increase from mild to severe disease but decline sharply in critically ill patients [78]. Notably, a positive correlation has been reported between elevated levels of CXCR3^+^ Tregs and disease severity [113]. Moreover, Tregs from patients with severe COVID-19 exhibit increased expression of Th1-associated markers, including CXCR3, GZMK, IL12RB1, and T-bet [112].

Currently, the role of Treg subset imbalance and altered functional activity in post-COVID-19 syndrome remains an active area of investigation [114]. Importantly, disturbances in Treg composition have been shown to persist for up to six months, even in individuals who experienced only mild acute disease [115]. Our preliminary data indicate that alterations in the Treg subpopulation profile persist for up to one year after the acute phase [116]. These changes are associated with a decreased proportion of “naïve” or “thymic” Tregs and reduced concentrations of CD73^+^ Tregs in peripheral blood. Nonetheless, several studies have reported eventual restoration of Treg parameters to baseline levels [117,118].

Taken together, numerous studies highlight that long COVID is associated with systemic hyperactivation of inflammatory mediators, disruption of central autonomic regulation of circulation and respiration, dysregulation of haemostatic and anti-haemostatic balance, and broad immune dysregulation involving autoimmunity and altered immuno–neuroendocrine interactions [119]. The principal route of SARS-CoV-2 entry is through cells expressing angiotensin-converting enzyme 2 (ACE2), the distribution of which determines the susceptibility of various organs and tissues to viral infection. Reduced thymic activity diminishes the output of newly generated (“naïve”) T cells, impairing immune recovery after disease. Monitoring thymic activity is therefore crucial for assessing COVID-19 severity and progression. Adrenal and thyroid dysfunctions have also been documented in PCS [25,120], and some data suggest a protective role for oxytocin-mediated regulation [121]; however, the involvement of hypothalamic–pituitary and pineal neuroendocrine regulators in PCS remains insufficiently understood.

Disturbances in T follicular helper (Tfh) cell differentiation—particularly in Bcl-6–expressing subsets—lead to impaired antibody formation, while imbalances among Tfh subpopulations contribute to heightened autoimmune risk and post-COVID-19 complications. Persistent B-cell activation following COVID-19, characterised by elevated levels of activated B cells and plasma cells, is well documented. Abnormalities in B-cell phenotype, such as prominent CD19^+^, CD80^+^/CD86^+^, and CD69^+^ expression, may increase the likelihood of autoimmune reactivity. The expansion of autoreactive B cells raises the risk of post-infectious autoimmune disorders.

Regulatory T cells play a pivotal role in maintaining immune homeostasis by controlling inflammation and preventing autoreactive responses [122]. COVID-19 disrupts the balance of Treg subpopulations, diminishing their regulatory capacity and contributing to the risk of chronic complications. Persistent disturbances in Treg subset composition have been observed up to one year after infection, influencing recovery and clinical outcomes.

Overall, available evidence depicts a complex network of immunological alterations after COVID-19, encompassing chronic inflammation, thymic dysfunction, B-cell and Tfh abnormalities, and persistent Treg imbalance. These changes contribute to increased risks of severe infection, prolonged symptomatology (“post-COVID”), autoimmune pathology, and recurrent infections.

## 5. Application of Artificial Intelligence and Machine Learning in Post-COVID-19 Syndrome Research

The first studies emphasizing the necessity of applying artificial intelligence (AI) for the analysis and prediction of the rapidly expanding data on the novel coronavirus infection appeared within the initial months of the pandemic. These early works anticipated a growing role for AI-based tools in the post-pandemic era. Nevertheless, the application of machine learning in post-COVID-19 syndrome (PCS) research remains fragmented and underexplored.

One of the most promising directions in current PCS investigations involves the study of autonomic cardiovascular dysfunction using heart rate variability (HRV) analysis combined with AI technologies. Shah et al. (2022) aimed to determine the prevalence of cardiovascular dysautonomia—including postural orthostatic tachycardia syndrome and orthostatic hypotension—among patients who had recovered from COVID-19, based on HRV assessment [110]. To achieve this objective, the authors developed and validated an AI model designed to identify the most informative time-domain HRV parameters obtained from short-term electrocardiographic (ECG) recordings, allowing differentiation between post-COVID-19 patients and healthy control subjects [123].

Using an interpretable AI framework (ShAP), the model was constructed to define HRV time-domain characteristics reflective of post-COVID-19 status. The findings demonstrated that patients with a history of SARS-CoV-2 infection frequently exhibited pronounced autonomic dysfunction of the cardiovascular system, manifested as significantly reduced HRV compared with healthy controls. The AI model effectively discriminated between post-COVID-19 individuals and healthy participants, confirming its diagnostic utility [110].

A systematic review encompassing 20 scientific publications selected studies employing innovative AI-based approaches. Investigations limited to conventional statistical analysis or theoretical discussions without applied AI results were excluded, as were publications issued before 2020 [101,124]. The review revealed that key trends in the application of AI to long COVID research involve the use of machine and deep learning methodologies, including ensemble-based models.

Jha et al. (2023) proposed a supervised learning strategy—Optimised XGBoost—based on the gradient boosting algorithm [125]. The primary objective was to predict the risk of post-COVID-19 pulmonary fibrosis in patients with severe disease within 90 days after hospital discharge. Model development utilised electronic medical records (EMRs) and high-resolution computed tomography (HRCT) data of the chest from 1175 patients, including 725 cases of pulmonary fibrosis and 450 normal controls. The results demonstrated high model performance: accuracy of 98%, precision of 99%, and sensitivity of 99%. The authors emphasised that their model represents the first system in the literature capable of integrating EMR and HRCT data for assessing post-COVID-19 pulmonary fibrosis risk, offering potential for reducing the likelihood of severe and potentially fatal outcomes [125].

### 5.1. Application of Artificial Intelligence Methods for the Detection and Prediction of Post-COVID-19 Complications

Recent studies demonstrate that the use of artificial intelligence (AI) algorithms—particularly interpretable models (ShAP), gradient boosting (Optimised XGBoost), and ensemble learning (stacking)—substantially improves the accuracy of diagnosis and prediction of post-COVID-19 complications. AI-driven approaches enable the integration of diverse clinical and biomedical datasets, providing a personalised framework for risk assessment and the development of preventive strategies in patients recovering from COVID-19 [67,126].

One of the most rapidly evolving areas in post-COVID-19 syndrome (PCS) research is the application of AI and machine learning (ML) techniques for diagnosis, risk stratification, and prediction of long-term consequences of COVID-19. A comprehensive analysis of the literature reveals that current approaches primarily rely on the integration of clinical records, electronic medical records (EMRs), biosignals, and imaging data, employing algorithms of interpretable AI, ensemble learning, and deep neural networks.

However, many existing models rely predominantly on clinical and demographic variables, with limited integration of immunological biomarkers.

Common limitations include small sample sizes, lack of external validation, risk of overfitting, and heterogeneity in outcome definitions, which constrain clinical applicability.

### 5.2. Identification of Patients with Long COVID Based on Electronic Health Record Data

Pfaff et al. (2022) utilised XGBoost models to identify patients with long COVID using the extensive N3C database, which includes data on over eight million individuals [127]. From a cohort of 1,793,604 adult patients, 97,995 with a confirmed COVID-19 diagnosis were selected. The analysis incorporated 924 features derived from 597 patients diagnosed with long COVID, including healthcare utilisation frequency, demographic variables, comorbidities, and medication use. The models were trained and validated on separate subsets (80/20% for hospitalised and 75/25% for non-hospitalised patients). The results demonstrated high area under the curve (AUC) values—0.92 for the total cohort, 0.90 for hospitalised patients, and 0.85 for outpatients. The main predictors identified through Shapley values included healthcare visit frequency, age, dyspnoea, and comorbid conditions. The XGBoost model thus proved to be highly effective for stratifying patients with PCS [127].

### 5.3. Prediction of Post-COVID-19 Cardiovascular Complications

Gupta et al. (2022) proposed an ensemble ML model based on the stacking method to predict post-COVID-19 cardiovascular complications [128]. The model was initially trained on data from 180 COVID-19 patients, including age, disease severity, hospitalisation duration, and symptoms during and after infection. After expanding the dataset to 4700 records and conducting cross-validation, the model’s performance was compared with traditional learning algorithms such as decision trees, random forests, support vector machines, and artificial neural networks [128].

The ensemble model achieved a prediction accuracy of 93.23%, with minimal root mean square error (RMSE = 0.32) and mean absolute error (MAE = 0.23), confirming its robustness and diagnostic reliability. Compared with classical ML methods, the proposed approach provided a more consistent risk assessment for cardiovascular complications in post-COVID-19 patients [128].

### 5.4. Prediction of Long COVID Based on Vital Signs and Physiological Parameters

Jiang et al. (2023) examined the relationship between vital signs—including oxygen saturation, heart rate, and systolic and diastolic blood pressure—and the risk of long COVID in hospitalised patients [129]. Data from the first seven days of admission in the N3C cohort were analysed. Predictive models based on XGBoost, convolutional neural networks (CNNs), and long short-term memory (LSTM) networks were employed to analyse multidimensional time-series data of physiological parameters. Model performance, evaluated using fivefold cross-validation, demonstrated high predictive accuracy [129].

Hill et al. (2022) conducted a retrospective case–control study across 31 US healthcare systems (N3C) to identify risk factors associated with long COVID [130]. Patients with confirmed SARS-CoV-2 infection between March and December 2020 were included. Demographics, comorbidities, pharmacotherapy, and acute disease manifestations were analysed. The comparison between 8325 PCS patients and 41,625 controls employed logistic regression, random forest, and XGBoost models, achieving AUC values of 0.73 and 0.69, respectively. The most significant risk factors were older age, disease severity, and pre-existing chronic conditions [130].

Sudre et al. (2021) applied a random forest algorithm for early differentiation between short-term and long COVID using symptom data collected through mobile applications with user self-reporting. The sample included 2149 individuals [131]. The presence of more than five symptoms during the first week of illness was significantly associated with an increased risk of developing long COVID. The model achieved an AUC of 75.9% on day seven of observation, confirming the feasibility of early prediction of disease duration based on clinical presentation [131,132].

### 5.5. Machine Learning and Statistical Analysis Methods in the Study of Long COVID Risk Factors

Modern approaches to analysing risk factors and predicting the long-term course of COVID-19 increasingly incorporate methods of deep learning and regression modelling. The most significant recent studies demonstrate the application of hybrid neural network architectures alongside classical statistical models for processing clinical, epidemiological, and imaging data.

However, evidence comparing their performance remains limited, and superiority over traditional approaches is not consistently demonstrated.

### 5.6. Deep Learning

The use of hybrid neural networks that combine recurrent and convolutional architectures has shown high efficacy in analysing longitudinal medical data. Sengupta et al. (2022) employed a model based on a bidirectional long short-term memory network (BiLSTM) combined with a one-dimensional convolutional neural network (1D CNN) to analyse historical diagnostic code data from the N3C repository. The study aimed to determine the likelihood of developing long COVID in individual patients based on chronologically ordered diagnostic codes recorded within 45 days following the initial positive test or clinical diagnosis of COVID-19 [123].

To interpret the contribution of individual diagnoses to the model’s final decision, the authors used the Gradient-weighted Class Activation Mapping (Grad-CAM) technique, which identifies the most influential diagnostic codes. Diagnoses with the highest activation values were regarded as key predictors of prolonged disease. The model was trained on a dataset split into training, validation, and testing subsets (75%, 15%, and 10%, respectively) and achieved a mean AUC of 0.7048 under threefold stratified cross-validation, despite substantial data imbalance [43].

In contrast, Subramanian et al. (2022) utilised convolutional neural networks (CNNs) to analyse high-resolution chest computed tomography (HRCT) images. The objective was to perform binary classification of images to identify regions of pulmonary involvement in COVID-19 patients with different recovery durations [131,133]. Two architectures—VGG16 and ResNet-50 —were trained on a dataset comprising 925 HRCT images divided into training (585), validation (65), and testing (275) subsets [134]. The best-performing model achieved an accuracy of 97.13%, while the introduction of a modified loss function combining Dirichlet and binary cross-entropy losses further improved accuracy to 98.2%. In the final stage, a U-Net–based segmentation model was developed, achieving 99.40% accuracy in identifying COVID-19–affected lung regions [133].

### 5.7. Regression Models

Beyond deep learning methods, substantial results have been achieved using regression-based approaches. Binka et al. (2022) proposed an Elastic Net regression model to identify long COVID cases using administrative healthcare data from the province of British Columbia, Canada. Input features included demographic characteristics, chronic comorbidities, COVID-19–related variables, and symptoms recorded between 28 and 183 days after diagnosis. The model was trained using 10-fold cross-validation and demonstrated strong diagnostic performance, with a sensitivity of 86%, specificity of 93%, and AUC of 0.93. Among 141,381 individuals who had recovered from COVID-19, 25,220 were classified as having features consistent with long COVID [135].

Moreno-Pérez et al. (2021) employed multiple logistic regression to assess acute-phase risk factors associated with the development of post-COVID-19 syndrome. The study included patients who had recovered from COVID-19, with follow-up evaluations performed 10–14 weeks post-infection. Analysis of clinical, laboratory, and pulmonary function data showed that post-COVID-19 syndrome was present in approximately half of the participants, whereas persistent radiological or spirometric abnormalities were observed in fewer than 25%. Multiple logistic regression analysis revealed no independent predictors significantly associated with the development of post-COVID-19 syndrome [136].

### 5.8. Other Approaches: Topic Modelling, Data Processing, and Text Mining

A study proposed a machine learning and topic-modelling-based framework to identify subcategories of long COVID based on new-onset medical conditions arising during the post-acute phase (30–180 days after COVID-19). The study utilised two large cohort databases—INSIGHT and OneFlorida+—comprising 20,881 and 13,724 patients, respectively. Based on more than 137 symptoms and conditions recorded within this period, the authors constructed 137-dimensional binary patient vectors and applied topic modelling to reduce feature dimensionality [98,105].

### 5.9. Role of Text Mining in the Diagnosis and Management of Post-COVID-19 Syndrome

Miao, Last, and Litvak (2022) applied a BERT-based natural language processing (NLP) approach to analyse 30,327 Twitter posts related to long COVID symptoms [137]. The study aimed to track the temporal dynamics of symptoms and their distribution by sex, age, geography, and symptom persistence [137]. Two datasets (May–December 2020 and October 2021) were used to analyse the evolution of symptom descriptions. The BERT classifier achieved 89% accuracy in analysing demographic categories and 95% in symptom identification [137].

Similarly, Zhu et al. (2022) employed three pre-trained BERT models to analyse unstructured clinical notes from patients with chronic COVID-19 symptoms [88]. The goal was to identify patients exhibiting long COVID features in outpatient records within 30–365 days post-diagnosis. Based on data from 719 patients and using 5-fold cross-validation, the model achieved a sensitivity of 0.88 when predicting at the level of individual clinical notes [138].

Scarpino et al. (2022) conducted a comparative analysis of topic-modelling approaches—Latent Dirichlet Allocation (LDA) and BERTopic—applied to textual data describing patient experiences of long COVID and healthcare professionals’ or public perceptions shared via social media [119]. The BERTopic method, which integrates transformer-based embeddings with contextually weighted TF-IDF (c-TF-IDF), outperformed LDA, correctly clustering 97.26% of documents and achieving an overall classification accuracy of 91.97% [44].

### 5.10. Alternative Analytical Approaches to Long COVID

Unlike classification models, McCorkell L et al. (2022) utilised the Apriori association rule mining algorithm to identify patterns among long COVID symptoms using Twitter data (May 2020–December 2021) [139]. The objective was to explore symptom co-occurrence and determine the most frequent combinations. The most prevalent symptoms were cognitive impairment (“brain fog”), fatigue, and respiratory problems. Furthermore, it was found that 77% of patients reporting taste disturbances also experienced anosmia [139].

Wang et al. (2021) developed a post-COVID-19 symptom lexicon (PCLS-Lex) based on clinical records from patients assessed between days 51 and 110 following a positive COVID-19 test. Using NLP and the Unified Medical Language System (UMLS), the model extracted the most frequent symptoms, including pain, anxiety, depression, fatigue, dyspnoea, myalgia, headache, nausea, and gastroesophageal reflux. The model achieved a precision of 94% and recall of 84% on data from 23,505 patients [78,140].

Banda et al. (2021) combined machine learning and NLP with expert clinical validation to analyse 296,154 tweets containing self-reported COVID-19 symptoms [126]. The resulting temporal analysis of symptoms over a 150-day post-infection period allowed the construction of individual symptom timelines and identification of symptom clusters [126].

Déguilhem et al. (2022) explored long COVID symptom profiles using data from French social media platforms (Twitter and Doctissimo forum). Applying Biterm Topic Modelling (BTM) and hierarchical clustering, the authors identified three common symptom clusters: fatigue–dyspnoea (35.3%), fatigue–anxiety (22.5%), and fatigue–headache (17.3%) [141]. The study also highlighted unmet patient needs related to daily activity limitations (Table 2).

Collectively, the evidence indicates that AI methodologies—including XGBoost, random forests, ensemble learning, and neural networks—are highly effective for diagnosing and predicting long-term outcomes of COVID-19. The application of AI to physiological data, electronic health records, and symptom-based datasets enhances diagnostic precision and supports the development of personalised prevention and rehabilitation strategies for post-COVID-19 patients. A meta-analysis based on the PRISMA framework summarised studies investigating COVID-19–induced acute respiratory distress syndrome (ARDS), identifying eight pathophysiological stages of the condition [142]. Artificial intelligence (AI) specialists have developed a range of intelligent tools for automated computer-aided diagnosis across imaging modalities such as lung computed tomography (CT), chest radiography, and lung ultrasonography. These tools assist pulmonologists and intensivists in promptly detecting viral involvement, classifying pneumonia subtypes, and assessing the severity of pulmonary damage in COVID-19 patients, emphasising the need for rapid and accurate imaging analysis [132].

Thus, contemporary AI models for ARDS diagnosis can be enhanced by integrating comorbidities as independent predictive factors. Clinical systems must incorporate model validation and verification processes to ensure reliability, stability, reproducibility, and generalisability, particularly when applied in pulmonology, intensive care, and radiology.

### 5.11. Bridging Immunopathology and Predictive Modelling

Although numerous immunological abnormalities have been described in PCS, only a limited number of biomarkers have been directly incorporated into predictive AI/ML models. Current prediction studies predominantly rely on demographic characteristics, clinical manifestations, physiological parameters, imaging findings, and electronic health records.

Among immunological variables already investigated in predictive frameworks are inflammatory markers (CRP, IL-6, TNF-α), lymphocyte counts and subsets, neutrophil-to-lymphocyte ratio, selected autoantibodies, and markers of immune activation. However, many biologically plausible biomarkers discussed in this review—including TREC/KREC-based indicators of thymic activity, detailed Tfh and Treg subpopulation profiles, cytokine autoantibodies, and markers of immune–neuroendocrine dysregulation—have not yet been systematically integrated into validated prediction models [17,24,94,143].

Therefore, these biomarkers should be considered promising candidates for future explainable AI and machine learning frameworks designed to improve risk stratification, phenotyping, and personalised management of post-COVID-19 syndrome.

## 6. Structured Qualitative Synthesis and Critical Comparison

The included studies demonstrate substantial heterogeneity in design, population characteristics, and outcome definitions. PCS was variably defined based on symptom duration (4 weeks vs. 12 weeks), clinical phenotype, or healthcare utilisation, limiting comparability.

Immunological studies predominantly employed observational designs and differed in biomarker panels, with inconsistent replication across cohorts. While thymic dysfunction and T-cell imbalance emerged as recurring findings, their quantitative assessment and clinical thresholds remain poorly standardised.

The AI/ML literature included in this review is characterised by important methodological constraints. Many studies rely on single-centre datasets, lack external validation, and are at risk of overfitting. In addition, issues such as class imbalance, data leakage, and inconsistent reporting of model calibration are frequently underdressed. The absence of benchmarking against conventional statistical models further limits interpretation of the true added value of AI-based approaches.

Risk of bias assessment using PROBAST indicated that a substantial proportion of prediction studies fall into moderate-to-high risk categories, particularly in the analysis domain. This reflects common limitations in model development, including inadequate handling of missing data, insufficient transparency in feature selection, and lack of prospective validation.

Potential publication bias should be considered, as studies reporting positive or high-performing AI models are more likely to be published. Similarly, the restriction to English-language publications may have led to the exclusion of relevant data.

The integration of immunopathological and computational findings remains largely conceptual. Few studies directly combine mechanistically grounded biomarkers with validated predictive models, highlighting a translational gap between biological insight and clinical implementation.

Overall, these limitations underscore the need for standardised definitions of PCS, large multicentre cohorts, rigorous validation of predictive models, and closer integration of systems immunology with explainable and clinically interpretable AI frameworks.

The AI/ML literature included in this review is characterised by important methodological constraints.

Many studies reported exceptionally high predictive performance (AUC > 0.90 or accuracy > 95%), raising concerns regarding potential overfitting, particularly in relatively small cohorts. External validation was absent in the majority of studies, limiting confidence in model generalisability across healthcare systems and populations. Class imbalance was frequently present because PCS cases constituted a minority of the analysed cohorts. Although several studies employed balancing techniques, reporting was often incomplete.

Calibration metrics were rarely reported. Consequently, even models with excellent discrimination may provide poorly calibrated risk estimates, restricting clinical implementation. Potential data leakage could not be excluded in several retrospective studies because feature extraction and outcome definition were performed within overlapping temporal windows. Only a minority of investigations directly compared AI approaches with conventional statistical models such as logistic regression. In many cases, improvements achieved by complex machine-learning methods were modest, suggesting that increased model complexity does not necessarily translate into superior clinical utility [76,144].

Risk of bias assessment using PROBAST indicated that a substantial proportion of prediction studies fall into moderate-to-high risk categories, particularly in the analysis domain. Importantly, the translational gap between immunological discoveries and predictive modelling remains considerable. While immunological studies consistently identify persistent cytokine dysregulation, autoimmunity, thymic dysfunction, and T-cell imbalance as central features of PCS, these mechanistically relevant biomarkers are rarely incorporated into AI-based prediction tools. Closing this gap represents one of the major priorities for future PCS research.

In addition, the reviewed literature demonstrates substantial heterogeneity across post-COVID-19 phenotypes. Particular attention should be paid to multisystem inflammatory syndrome in adults (MIS-A) and children (MIS-C), which represent severe post-infectious hyperinflammatory entities sharing several immunopathological features with PCS but differing in clinical presentation, timing, and severity. These conditions should therefore be regarded as related but distinct components within the broader spectrum of post-COVID-19 immune-mediated disorders.

### 6.1. Risk of Bias Assessment of AI/ML Prediction Models

Risk of bias assessment using PROBAST revealed that only a limited number of studies could be classified as having low-to-moderate overall risk of bias. The most frequent concerns were identified within the analysis domain, including inadequate handling of missing data, insufficient reporting of model calibration, lack of external validation, and potential overfitting [42,145].

Studies based on large multicentre electronic health record databases generally demonstrated lower risk in the participant and predictor domains. In contrast, investigations relying on social media data, self-reported symptoms, or highly selected cohorts exhibited increased risks related to participant selection and outcome ascertainment.

Overall, the analysis domain represented the principal source of bias across the AI/ML literature. These findings indicate that despite promising predictive performance, many currently available models require prospective validation and independent replication before routine clinical implementation.

### 6.2. Limitations

Several limitations should be considered when interpreting the findings of this review. Post-COVID syndrome remains a highly heterogeneous condition, and definitions varied considerably across studies. Some investigations defined PCS as symptoms persisting beyond four weeks after acute infection, whereas others used the twelve-week threshold recommended by international guidelines. This variability complicates direct comparison of study outcomes and may contribute to inconsistencies in reported associations.

Substantial heterogeneity was observed in study populations, follow-up durations, clinical phenotypes, and biomarker panels. Immunological investigations employed different laboratory methodologies and evaluated distinct combinations of cytokines, autoantibodies, lymphocyte subsets, and inflammatory markers, limiting cross-study comparability and preventing quantitative pooling of results.

Although thymic dysfunction, T-cell imbalance, B-cell activation, autoimmunity, and chronic inflammation emerged as recurring themes, many immunological findings have not yet been replicated across large independent cohorts. Consequently, the clinical utility of several proposed biomarkers remains uncertain [146,147].

The AI and machine-learning literature demonstrated important methodological limitations. Many prediction models were developed using single-centre or retrospective datasets, frequently lacked external validation, and often relied on relatively small sample sizes. Several studies provided limited information regarding calibration, feature selection procedures, handling of missing data, and model reproducibility.

Common risks identified through PROBAST assessment included potential overfitting, class imbalance, data leakage, inadequate reporting of validation procedures, and insufficient comparison with conventional statistical approaches. These factors may lead to overestimation of model performance and limit generalisability to real-world clinical settings.

Publication bias cannot be excluded. Studies reporting positive findings, significant biomarker associations, or high-performing AI models are more likely to be published than studies with negative or inconclusive results. This may have contributed to an overly optimistic representation of predictive performance.

Despite efforts to conduct a comprehensive search, language and database restrictions may have resulted in omission of relevant studies published outside the indexed literature.

A major translational gap remains between immunopathological research and AI-based predictive modelling. While numerous studies have identified biologically plausible immune biomarkers, relatively few prediction models have incorporated mechanistically validated immunological variables. Consequently, the integration of systems immunology with explainable and clinically interpretable AI frameworks remains an important area for future research.

Investigations should prioritize standardized PCS definitions, multicentre prospective cohorts, harmonized biomarker assessment, rigorous external validation of predictive models, and closer integration of immunological mechanisms with clinically deployable AI systems.

## 7. Conclusions

Post-COVID-19 syndrome represents a multifactorial condition arising from the interplay of immunological, neuroendocrine, and inflammatory processes that persist beyond the acute phase of SARS-CoV-2 infection. Central pathogenic mechanisms include thymic impairment, reduced naïve T-cell output, sustained cytokine dysregulation, and potential reactivation of latent viral infections. These alterations may contribute to the development or exacerbation of chronic inflammatory and granulomatous diseases, as evidenced by increased TNF-α activity and the documented emergence of sarcoid-like granulomas following COVID-19.

In parallel, the rapid expansion of AI and ML research in the post-COVID-19 domain demonstrates the growing methodological capacity to analyze large-scale biomedical datasets and uncover clinically relevant patterns. Machine learning methods—ranging from gradient-boosting models and ensemble approaches to deep neural networks—have shown high performance in identifying long-COVID patients, predicting cardiovascular and pulmonary complications, and analysing physiological signals. NLP-based tools further enhance the extraction of symptomatic and diagnostic information from clinical notes and patient-generated data, enabling the identification of symptom clusters and long-COVID phenotypes.

The convergence of immunopathological insights with advanced computational methodologies underscores the necessity of integrated, data-driven approaches in the study and management of PCS. Future research should focus on external validation of AI models, harmonisation of clinical definitions, and incorporation of immunological biomarkers to improve model generalisability and translational potential.

## Figures and Tables

**Table 1:** Key autoantibodies in PCS/PASC: mechanisms and clinical associations.

Autoantibody Target	Proposed Mechanism	Affected Pathway	Reported Clinical Phenotypes
GPCRs (e.g., β2-adrenergic, M2 muscarinic receptors)	Functional receptor modulation (agonistic/antagonistic activity) leading to autonomic dysregulation	Autonomic nervous system imbalance	POTS, tachycardia, orthostatic intolerance, fatigue
ACE2	Interference with SARS-CoV-2 receptor and RAS signalling	Renin–angiotensin system dysregulation	Endothelial dysfunction, vascular symptoms
Phospholipids (e.g., cardiolipin, β2-glycoprotein I)	Prothrombotic state via endothelial activation and coagulation cascade	Coagulation and vascular inflammation	Microthrombosis, stroke risk, pulmonary embolism
Type I interferons	Neutralisation of antiviral signalling	Impaired innate immune response	Severe initial COVID-19, persistent viral reservoirs
Nuclear antigens (ANA, anti-dsDNA)	Loss of self-tolerance and systemic autoimmunity	Adaptive immune dysregulation	Lupus-like manifestations, fatigue, arthralgia
Endothelial cell antigens	Endothelial injury and chronic inflammation	Vascular dysfunction	Microvascular damage, chronic fatigue, dyspnoea
Neuronal antigens	Neuroinflammation and synaptic dysfunction	Central nervous system involvement	Cognitive impairment (“brain fog”), headaches
Cytokines/immune mediators	Dysregulation of cytokine signalling networks	Chronic inflammation	Persistent systemic inflammatory symptoms

**Table 2:** Prediction of post-COVID-19.

Author, Year	Data	Methods	Results	
Pfaff et al. (2022) [127]	N3C database; 1,793,604 adults (97,995 COVID-positive)	XGBoost, SHAP interpretability	Identification of long COVID patients using EHR data	AUC = 0.92 (overall); 0.90 (hospitalised); 0.85 (outpatient)
Gupta et al. (2022) [128]	Clinical data from 180 COVID-19 patients (expanded to 4700 records)	Stacking ensemble (Decision Tree, Random Forest, SVM, ANN)	Prediction of post-COVID-19 cardiovascular complications	Accuracy = 93.23%; RMSE = 0.32; MAE = 0.23
Jiang et al. (2023) [129]	N3C cohort, 7-day vitals (SpO_2_, HR, BP)	XGBoost, CNN, LSTM	Prediction of long COVID based on physiological time series	High predictive accuracy (cross-validation, 5-fold)
Hill et al. (2022) [130]	N3C; 8325 long COVID vs. 41,625 controls	Logistic regression, Random Forest, XGBoost	Risk factor identification for long COVID	AUC = 0.73 (RF); 0.69 (XGBoost)
Sudre et al. (2021) [132]	App-based symptom data; *n* = 2149	Random Forest	Early differentiation of short vs. long COVID	AUC = 75.9%; ≥5 symptoms in week 1 → high long-COVID risk
Zhang H. et al. (2023) [87]	HRCT lung images	CNN (VGG16, ResNet50, U-Net)	Detection of residual pulmonary changes after COVID-19	Accuracy = 98.5%; Dice = 0.91
Miao et al. (2022) [137]	Multi-centre CT dataset	3D CNN + Transfer Learning	Lung fibrosis risk prediction post-COVID-19	AUC = 0.94
Sengupta et al. (2022) [123]	N3C; diagnostic codes	BiLSTM + 1D CNN	Long COVID prediction via sequential code embedding	AUC = 0.7048
Zhu et al. (2023) [138]	EHR + laboratory data	Optimised Gradient Boosting (XGBoost)	Prediction of post-acute COVID-19 sequelae	AUC = 0.91

## Data Availability

The original contributions presented in the study are included in the article, further inquiries can be directed to the corresponding author.

## References

[1] Soriano J.B., Murthy S., Marshall J.C., Relan P., Diaz J.V., WHO Clinical Case Definition Working Group on Post-COVID-19 Condition (2022). A clinical case definition of post-COVID-19 condition by a Delphi consensus. Lancet Infect. Dis..

[2] Woodruff M.C., Ramonell R.P., Haddad N.S., Anam F.A., Rudolph M.E., Walker T.A., Truong A.D., Dixit A.N., Han J.E., Cabrera-Mora M. (2022). Dysregulated naive B cells and de novo autoreactivity in severe COVID-19. Nature.

[3] Youinou P., Renaudineau Y. (2007). The paradox of CD5-expressing B cells in systemic lupus erythematosus. Autoimmun. Rev..

[4] Davis H.E., McCorkell L., Vogel J.M., Topol E.J. (2023). Long COVID: Major findings, mechanisms and recommendations. Nat. Rev. Microbiol..

[5] Malkova A.M., Kudryavtsev I.V., Starshinova A.A., Kudlay D.A., Zinchenko Y.S., Glushkova A., Yablonskiy P., Shoenfeld Y. (2021). Post COVID-19 syndrome in patients with asymptomatic/mild form. Pathogens.

[6] Makarov I., Mayrina S., Makarova T., Karonova T., Starshinova A., Kudlay D., Mitrofanova L. (2023). Morphological Changes in the Myocardium of Patients with Post-Acute Coronavirus Syndrome: A Study of Endomyocardial Biopsies. Diagnostics.

[7] Rodriguez-Sanchez I., Rodriguez-Mañas L., Laosa O. (2022). Long COVID-19: The need for an interdisciplinary approach. Clin. Geriatr. Med..

[8] Bohmwald K. (2024). Immunological pathways in post-COVID-19 condition. Front. Immunol..

[9] Peluso M.J., Deeks S.G. (2024). Mechanisms of long COVID and the path toward therapeutics. Cell.

[10] Ryabkova V.A., Churilov L.P. (2023). Disease course and pathogenesis of post-COVID-19 condition. Autoimmunity, COVID-19, Post-COVID-19 Syndrome and COVID-19 Vaccination.

[11] Skevaki C., Moschopoulos C.D., Fragkou P.C., Grote K., Schieffer E., Schieffer B. (2025). Long COVID: Pathophysiology, current concepts, and future directions. J. Allergy Clin. Immunol..

[12] Normatov M.G., Karev V.E., Kolobov A.V., Mayevskaya V.A., Ryabkova V.A., Utekhin V.J., Churilov L.P. (2023). Post-COVID Endocrine Disorders: Putative Role of Molecular Mimicry and Some Pathomorphological Correlates. Diagnostics.

[13] Basina A.A., Akhmetova A., Gavrilova N., Soprun L.A., Volovnikova V.A., Utekhin V.I., Churilov L.P. (2024). Small fiber neuropathy in the pathogenesis of post-COVID syndrome. Russ. Biomed. Res..

[14] Churilov L.P., Normatov M.G., Utekhin V.J. (2022). Molecular Mimicry between SARS-CoV-2 and Human Endocrinocytes: A Prerequisite of Post-COVID-19 Endocrine Autoimmunity?. Pathophysiology.

[15] Fedotkina T.V., Gurevich V.S., Muzalevskaya M.V., Normatov M.G., Churilov L.P. (2023). Molecular mimicry between autoantigens of human endotheliocytes and coronaviruses and promotion of atherogenesis. 9th International Congress of Pathophysiology and 5th Congress of Physiological Sciences of Serbia with International Participation: Final Program and Abstract Book, Belgrade.

[16] Gavrilova N., Soprun L., Lukashenko M., Ryabkova V., Fedotkina T.V., Churilov L.P., Shoenfeld Y. (2022). New Clinical Phenotype of the Post-COVID Syndrome: Fibromyalgia and Joint Hypermobility Condition. Pathophysiology.

[17] Talamini L., Fonseca D.L.M., Kanduc D., Chaloin O., Verdot C., Galmiche C., Dotan A., Filgueiras I.S., Borghi M.O., Meroni P.L. (2025). Long COVID-19 autoantibodies and their potential effect on fertility. Front. Immunol..

[18] Churilov L.P. (2009). O sistemnom podhode v obshchej patologii: Neobhodimost’ i principy bioinformatiki [On the Systemic Approach in General Pathology. Necessity and Principles of the Pathoinformatics]. Vestn. St. Petersburg Univ. Med..

[19] Sobolevskaia P., Kolobov A., Churilov L. (2023). Central nervous system impairments in COVID-19. Autoimmunity, COVID-19, Post-COVID-19 Syndrome and COVID-19 Vaccination.

[20] Mitrofanova L., Makarov I., Goncharova E., Makarova T., Starshinova A., Kudlay D., Shlaykhto E. (2023). High Risk of Heart Tumors after COVID-19. Life.

[21] Morita R., Schmitt N., Bentebibel S.E., Ranganathan R., Bourdery L., Zurawski G., Foucat E., Dullaers M., Oh S., Sabzghabaei N. (2011). Human blood CXCR5^+^CD4^+^ T cells are counterparts of T follicular cells and contain specific subsets that differentially support antibody secretion. Immunity.

[22] Gavrilova N.Y., Normatov M.G., Soprun L.A., Utekhin V.J., Fedotkina T.V., Churilov L.P. (2024). Autoantigens of Small Nerve Fibers and Human Coronavirus Antigens: Is There a Possibility for Molecular Mimicry?. Curr. Microbiol..

[23] Ryabkova V.A., Rubinskiy A.V., Marchenko V.N., Trofimov V.I., Churilov L.P. (2024). Similar Patterns of Dysautonomia in Myalgic Encephalomyelitis/Chronic Fatigue and Post-COVID-19 Syndromes. Pathophysiology.

[24] Schipperijn N., Wijesinghe M., Romo A., Brooks B. (2024). Postural Orthostatic Tachycardia Syndrome (POTS) as an Adverse Event to the Human Papilloma Virus (HPV) Vaccine and Its Relationship with Ehlers–Danlos Syndrome (EDS). Reports.

[25] Sobolevskaya P.A., Gvozdetskiy A.N., Kudryavtsev I.V., Chereshnev V.A., Churilov L.P. (2024). Subpopulation analysis of T-follicular helper cells (Tfh) in patients with Hashimoto’s thyroiditis and mental disorders. Bull. Ural Med. Acad. Sci..

[26] Jayavelu N.D., Samaha H., Wimalasena S.T., Hoch A., Gygi J.P., Gabernet G., Ozonoff A., Liu S., Milliren C.E., Levy O. (2025). Machine learning models predict long COVID outcomes based on baseline clinical and immunologic factors. medRxiv.

[27] Wacleche V.S., Goulet J.P., Gosselin A., Monteiro P., Soudeyns H., Fromentin R., Jenabian M.A., Vartanian S., Deeks S.G., Chomont N. (2016). New insights into the heterogeneity of Th17 subsets contributing to HIV-1 persistence during antiretroviral therapy. Retrovirology.

[28] Page M.J., McKenzie J.E., Bossuyt P.M., Boutron I., Hoffmann T.C., Mulrow C.D., Shamseer L., Tetzlaff J.M., Akl E.A., Brennan S.E. (2021). The PRISMA 2020 statement: An updated guideline for reporting systematic reviews. BMJ.

[29] Bistagnino F., Subramanian A., Tovani-Palone M.R. (2025). Navigating the (Post-) Pandemic Landscape: An Analysis of COVID-19’s Current Status and Future Implications. Disaster Med. Public Health Prep..

[30] Khakshooy A., Chiappelli F. (2024). Post-acute COVID-19 syndrome (PACS) linked cardiovascular symptoms. Bioinformation.

[31] Lang S.M., Schiffl H. (2025). Long-term renal consequences of COVID-19. Emerging evidence and unanswered questions. Int. Urol. Nephrol..

[32] Makarova Y.A., Ryabkova V.A., Salukhov V.V., Sagun B.V., Korovin A.E., Churilov L.P. (2023). Atherosclerosis, Cardiovascular Disorders and COVID-19: Comorbid Pathogenesis. Diagnostics.

[33] O’Neil L.J., Barrera-Vargas A., Sandoval-Heglund D., Merayo-Chalico J., Aguirre-Aguilar E., Aponte A.M., Ruiz-Perdomo Y., Gucek M., El-Gabalawy H., Fox D.A. (2020). Neutrophil-mediated carbamylation promotes articular damage in rheumatoid arthritis. Sci. Adv..

[34] Paulissen S.M., van Hamburg J.P., Dankers W., Lubberts E. (2015). The role and modulation of CCR6+ Th17 cell populations in rheumatoid arthritis. Cytokine.

[35] Batiha G.E., Al-Kuraishy H.M., Al-Gareeb A.I., Welson N.N. (2022). Pathophysiology of Post-COVID syndromes: A new perspective. Virol. J..

[36] Chadaga K., Prabhu S., Sampathila N., Chadaga R., Umakanth S., Bhat D., Shashi Kumar G.S. (2024). Explainable artificial intelligence approaches for COVID-19 prognosis prediction using clinical markers. Sci. Rep..

[37] Churilov L.P. (2020). Obshchaya Patofiziologiya s Osnovami Immunopatologii [General Pathophysiology with Fundamentals of Immunopathology].

[38] Starshinova A.A., Savchenko A.A., Borisov A., Kudryavtsev I., Rubinstein A., Dovgalyuk I., Kulpina A., Churilov L.P., Sobolevskaia P., Fedotkina T. (2025). Immunological Disorders: Gradations and the Current Approach in Laboratory Diagnostics. Pathophysiology.

[39] Xiao M., Zhang Y., Zhang S., Qin X., Xia P., Cao W., Jiang W., Chen H., Ding X., Zhao H. (2020). Antiphospholipid Antibodies in Critically Ill Patients with COVID-19. Arthritis Rheumatol..

[40] Hoheisel F., Fleischer K.M., Rubarth K., Sepúlveda N., Bauer S., Konietschke F., Kedor Peters C., Stein A.E., Wittke K., Seifert M. (2025). Exploratory study on autoantibodies to arginine-rich human peptides mimicking Epstein-Barr virus in women with post-COVID and myalgic encephalomyelitis/chronic fatigue syndrome. Front. Immunol..

[41] Osipov G.A., Verkhovtseva N.V. (2011). Study of human microecology by mass spectrometry of microbial markers. Benef. Microbes.

[42] Ryabkova V.A., Shoenfeld, Churilov L.P. (2021). COVID-19 and ABO blood groups. Isr. Med. Assoc. J..

[43] Schmitt N., Bentebibel S.E., Ueno H. (2014). Phenotype and functions of memory Tfh cells in human blood. Trends Immunol..

[44] Savchenko A.A., Tikhonova E., Kudryavtsev I., Kudlay D., Korsunsky I., Beleniuk V., Borisov A. (2022). TREC/KREC Levels and T and B Lymphocyte Subpopulations in COVID-19 Patients at Different Stages of the Disease. Viruses.

[45] di Filippo L., Franzese V., Santoro S., Doga M., Giustina A. (2024). Long COVID and pituitary dysfunctions: A bidirectional relationship?. Pituitary.

[46] Hadi Y.B., Lakhani D.A., Naqvi S.F.Z., Singh S., Kupec J.T. (2021). Outcomes of SARS-CoV-2 infection in patients with pulmonary sarcoidosis: A multicenter retrospective research network study. Respir. Med..

[47] Sanz I., Wei C., Lee F.E., Anolik J. (2008). Phenotypic and functional heterogeneity of human memory B cells. Semin. Immunol..

[48] Khadzhieva M.B., Kalinina E.V., Larin S.S., Sviridova D.A., Gracheva A.S., Chursinova J.V., Stepanov V.A., Redkin I.V., Avdeikina L.S., Rumyantsev A.G. (2021). TREC/KREC Levels in Young COVID-19 Patients. Diagnostics.

[49] Sasikumar S., Unniappan S. (2024). SARS-CoV-2 Infection and the Neuroendocrine System. Neuroendocrinology.

[50] Rosichini M., Bordoni V., Silvestris D.A., Mariotti D., Matusali G., Cardinale A., Zambruno G., Condorelli A.G., Flamini S., Genah S. (2023). SARS-CoV-2 infection of thymus induces loss of function that correlates with disease severity. J. Allergy Clin. Immunol..

[51] Ryan F.J., Hope C.M., Masavuli M.G., Lynn M.A., Mekonnen Z.A., Yeow A.E.L., Garcia-Valtanen P., Al-Delfi Z., Gummow J., Ferguson C. (2022). Long-term perturbation of the peripheral immune system months after SARS-CoV-2 infection. BMC Med..

[52] De Bruyn A., Verellen S., Bruckers L., Geebelen L., Callebaut I., De Pauw I., Stessel B., Dubois J. (2022). Secondary infection in COVID-19 critically ill patients: A retrospective single-center evaluation. BMC Infect. Dis..

[53] Gold J.E., Okyay R.A., Licht W.E., Hurley D.J. (2021). Investigation of Long COVID Prevalence and Its Relationship to Epstein-Barr Virus Reactivation. Pathogens.

[54] Yasar K., Arzu G., Akif O.M., Şenay A., Zeynep C.H., Pınar F., Banu S. (2022). Is there a relationship between COVID-19 and sarcoidosis? A case report. Arch. Pulmonol. Respir. Care.

[55] Shcherbak S.G., Anisenkova A.Y.U., Mosenko S.V., Puzankova E.V., Mamaeva O.P., Vologzhanin D.A., Gavrilova N.Y.U., Ryabkova V.A., Churilov L.P., Ratnikov E.D. (2024). Dolgij COVID. Sovremennoe sostoyanie problemy i perspektivy izucheniya i lecheniya. Chast’ 2 [Long COVID. State of Art and Perspectives of Studies and Treatment. Part II]. Clin. Pathophysiol..

[56] Gil-Manso S., Blanco I.M., López-Esteban R., Carbonell D., López-Fernández L.A., West L., Correa-Rocha R., Pion M. (2022). Comprehensive Flow Cytometry Profiling of the Immune System in COVID-19 Convalescent Individuals. Front. Immunol..

[57] Halim L., Romano M., McGregor R., Correa I., Pavlidis P., Grageda N., Hoong S.J., Yuksel M., Jassem W., Hannen R.F. (2017). An Atlas of Human Regulatory T Helper-like Cells Reveals Features of Th2-like Tregs that Support a Tumorigenic Environment. Cell Rep..

[58] Castleman M.J., Stumpf M.M., Therrien N.R., Smith M.J., Lesteberg K.E., Palmer B.E., Maloney J.P., Janssen W.J., Mould K.J., Beckham J.D. (2022). SARS-CoV-2 infection relaxes peripheral B cell tolerance. J. Exp. Med..

[59] Hanley P., Sutter J.A., Goodman N.G., Du Y., Sekiguchi D.R., Meng W., Rickels M.R., Naji A., Luning Prak E.T. (2017). Circulating B cells in type 1 diabetics exhibit fewer maturation-associated phenotypes. Clin. Immunol..

[60] Yates F.E., Goldenberger R.F., Yamamoto K. (1983). Systems analysis of hormone action. Biological Regulation and Development.

[61] Rubinstein A., Kudryavtsev I., Arsentieva N., Korobova Z.R., Isakov D., Totolian A.A. (2024). CXCR3-Expressing T Cells in Infections and Autoimmunity. Front. Biosci. (Landmark Ed.).

[62] Bohnhorst J.O., Thoen J.E., Natvig J.B., Thompson K.M. (2001). Significantly depressed percentage of CD27+ (memory) B cells among peripheral blood B cells in patients with primary Sjögren’s syndrome. Scand. J. Immunol..

[63] Chicco D., Jurman G. (2020). Machine Learning Can Predict Survival of Patients with Heart Failure from Serum Creatinine and Ejection Fraction Alone. BMC Med. Inform. Decis. Mak..

[64] Vick S.C., Frutoso M., Mair F., Konecny A.J., Greene E., Wolf C.R., Logue J.K., Franko N.M., Boonyaratanakornkit J., Gottardo R. (2021). A regulatory T cell signature distinguishes the immune landscape of COVID-19 patients from those with other respiratory infections. Sci. Adv..

[65] Dhaeze T., Peelen E., Hombrouck A., Peeters L., Van Wijmeersch B., Lemkens N., Lemkens P., Somers V., Lucas S., Broux B. (2015). Circulating Follicular Regulatory T Cells Are Defective in Multiple Sclerosis. J. Immunol..

[66] Duhen T., Duhen R., Lanzavecchia A., Sallusto F., Campbell D.J. (2012). Functionally distinct subsets of human FOXP3+ Treg cells that phenotypically mirror effector Th cells. Blood.

[67] Abarca-Zabalía J., González-Jiménez A., Calle-Rubio M., López-Pastor A.R., Fariña T., Ramos-Acosta C., Anguita E., Urcelay E., Espino-Paisán L. (2023). Alterations in the immune system persist after one year of convalescence in severe COVID-19 patients. Front. Immunol..

[68] Garmendia J.V., García A.H., De Sanctis C.V., Hajdúch M., De Sanctis J.B. (2022). Autoimmunity and Immunodeficiency in Severe SARS-CoV-2 Infection and Prolonged COVID-19. Curr. Issues Mol. Biol..

[69] Jiang S., Loomba J., Sharma S., Brown D., RECOVER Consortium, N3C Consortium (2022). Vital Measurements of Hospitalized COVID-19 Patients as a Predictor of Long COVID: An EHR-based Cohort Study from the RECOVER Program in N3C. arXiv.

[70] Acosta-Ampudia Y., Monsalve D.M., Rojas M., Rodríguez Y., Gallo J.E., Salazar-Uribe J.C., Santander M.J., Cala M.P., Zapata W., Zapata M.I. (2021). COVID-19 convalescent plasma composition and immunological effects in severe patients. J. Autoimmun..

[71] Gensous N., Charrier M., Duluc D., Contin-Bordes C., Truchetet M.E., Lazaro E., Duffau P., Blanco P., Richez C. (2018). T Follicular Helper Cells in Autoimmune Disorders. Front. Immunol..

[72] Kumar R., Kumar A., Saroj U., Kumar M., Singh S.K., Kumar A., Singh P.K., Munda P.K., Choudhary A.K., Farheen Z. (2022). A cross-sectional study of clinical and laboratory characteristics of systemic lupus erythematosus in tribal region of Jharkhand at RIMS, Ranchi. J. Fam. Med. Prim. Care.

[73] Chang S.E., Feng A., Meng W., Apostolidis S.A., Mack E., Artandi M., Barman L., Bennett K., Chakraborty S., Chang I. (2021). New-onset IgG autoantibodies in hospitalized patients with COVID-19. Nat. Commun..

[74] Bastard P., Rosen L.B., Zhang Q., Michailidis E., Hoffmann H.H., Zhang Y., Dorgham K., Philippot Q., Rosain J., Béziat V. (2020). Autoantibodies against type I IFNs in patients with life-threatening COVID-19. Science.

[75] Ayoglu B., Donato M., Furst D.E., Crofford L.J., Goldmuntz E., Keyes-Elstein L., James J., Macwana S., Mayes M.D., McSweeney P. (2023). Characterising the autoantibody repertoire in systemic sclerosis following myeloablative haematopoietic stem cell transplantation. Ann. Rheum. Dis..

[76] Price J.V., Haddon D.J., Kemmer D., Delepine G., Mandelbaum G., Jarrell J.A., Gupta R., Balboni I., Chakravarty E.F., Sokolove J. (2013). Protein microarray analysis reveals BAFF-binding autoantibodies in systemic lupus erythematosus. J. Clin. Investig..

[77] Wagner C., Griesel M., Mikolajewska A., Mueller A., Nothacker M., Kley K., Metzendorf M.I., Fischer A.L., Kopp M., Stegemann M. (2022). Systemic corticosteroids for the treatment of COVID-19: Equity-related analyses and update on evidence. Cochrane Database Syst. Rev..

[78] Wang E.Y., Mao T., Klein J., Dai Y., Huck J.D., Jaycox J.R., Liu F., Zhou T., Israelow B., Wong P. (2021). Diverse functional autoantibodies in patients with COVID-19. Nature.

[79] Li Y., Jia R., Liu Y., Tang S., Ma X., Shi L., Zhao J., Hu F., Li Z. (2020). Antibodies against carbamylated vimentin exist in systemic lupus erythematosus and correlate with disease activity. Lupus.

[80] Gagiannis D., Steinestel J., Hackenbroch C., Schreiner B., Hannemann M., Bloch W., Umathum V.G., Gebauer N., Rother C., Stahl M. (2020). Clinical, Serological, and Histopathological Similarities Between Severe COVID-19 and Acute Exacerbation of Connective Tissue Disease-Associated Interstitial Lung Disease (CTD-ILD). Front. Immunol..

[81] Grygiel-Górniak B., Rogacka N., Puszczewicz M. (2018). Antinuclear antibodies in healthy people and non-rheumatic diseases—Diagnostic and clinical implications. Reumatologia.

[82] Basic-Jukic N., Pavlisa G., Sremec N.T., Juric I., Ledenko R., Rogic D., Jelakovic B. (2023). Autoantibodies in COVID-19, a possible role in the pathogenesis of the disease. Ther. Apher. Dial..

[83] Jeong H., Baek A.R., Park S.W., Kim T., Choo E.J., Jeon C.H. (2023). Rheumatoid factor is associated with severe COVID-19. Int. J. Rheum. Dis..

[84] Pascolini S., Granito A., Muratori L., Lenzi M., Muratori P. (2021). Coronavirus disease associated immune thrombocytopenia: Causation or correlation?. J. Microbiol. Immunol. Infect..

[85] Pascolini S., Vannini A., Deleonardi G., Ciordinik M., Sensoli A., Carletti I., Veronesi L., Ricci C., Pronesti A., Mazzanti L. (2021). COVID-19 and Immunological Dysregulation: Can Autoantibodies be Useful?. Clin. Transl. Sci..

[86] World Health Organization (2024). Post COVID-19 Condition (Long COVID): Fact Sheet.

[87] Vasichkina E., Alekseeva D., Karev V., Podyacheva E., Kudryavtsev I., Glushkova A., Starshinova A.Y., Kudlay D., Starshinova A. (2023). Cardiac Involvement in Children Affected by COVID-19: Clinical Features and Diagnosis. Diagnostics.

[88] Zhu Y., Mahale A., Peters K., Mathew L., Giuste F., Anderson B., Wang M.D. (2022). Using natural language processing on free-text clinical notes to identify patients with long-term COVID effects. Proceedings of the 13th ACM International Conference on Bioinformatics, Computational Biology and Health Informatics.

[89] Zhang Y., Xiao M., Zhang S., Xia P., Cao W., Jiang W., Chen H., Ding X., Zhao H., Zhang H. (2020). Coagulopathy and Antiphospholipid Antibodies in Patients with COVID-19. N. Engl. J. Med..

[90] Zuo Y., Estes S.K., Ali R.A., Gandhi A.A., Yalavarthi S., Shi H., Sule G., Gockman K., Madison J.A., Zuo M. (2020). Prothrombotic autoantibodies in serum from patients hospitalized with COVID-19. Sci. Transl. Med..

[91] Anderson A.C., Joller N., Kuchroo V.K. (2016). Lag-3, Tim-3, and TIGIT: Co-inhibitory Receptors with Specialized Functions in Immune Regulation. Immunity.

[92] Crotty S. (2019). T Follicular Helper Cell Biology: A Decade of Discovery and Diseases. Immunity.

[93] Wiech M., Chroscicki P., Swatler J., Stepnik D., De Biasi S., Hampel M., Brewinska-Olchowik M., Maliszewska A., Sklinda K., Durlik M. (2022). Remodeling of T Cell Dynamics During Long COVID Is Dependent on Severity of SARS-CoV-2 Infection. Front. Immunol..

[94] Tangye S.G., Ma C.S., Brink R., Deenick E.K. (2013). The good, the bad and the ugly—TFH cells in human health and disease. Nat. Rev. Immunol..

[95] Qi J., Liu C., Bai Z., Li X., Yao G. (2023). T follicular helper cells and T follicular regulatory cells in autoimmune diseases. Front. Immunol..

[96] Vijayakumar B., Boustani K., Ogger P.P., Papadaki A., Tonkin J., Orton C.M., Ghai P., Suveizdyte K., Hewitt R.J., Desai S.R. (2022). Immuno-proteomic profiling reveals aberrant immune cell regulation in the airways of individuals with ongoing post-COVID-19 respiratory disease. Immunity.

[97] Laidlaw B.J., Ellebedy A.H. (2022). The germinal centre B cell response to SARS-CoV-2. Nat. Rev. Immunol..

[98] Zhang J., Wu Q., Liu Z., Wang Q., Wu J., Hu Y., Bai T., Xie T., Huang M., Wu T. (2021). Spike-specific circulating T follicular helper cell and cross-neutralizing antibody responses in COVID-19-convalescent individuals. Nat. Microbiol..

[99] Kalfaoglu B., Almeida-Santos J., Tye C.A., Satou Y., Ono M. (2021). T-cell dysregulation in COVID-19. Biochem. Biophys. Res. Commun..

[100] Kaneko N., Kuo H.-H., Boucau J., Farmer J.R., Allard-Chamard H., Mahajan V.S., Piechocka-Trocha A., Lefteri K., Osborn M., Bals J. (2020). Loss of Bcl-6-Expressing T Follicular Helper Cells and Germinal Centers in COVID-19. Cell.

[101] Ahmad I., Amelio A., Merla A., Scozzari F. (2024). A survey on the role of artificial intelligence in managing Long COVID. Front. Artif. Intell..

[102] Golovkin A., Kalinina O., Bezrukikh V., Aquino A., Zaikova E., Karonova T., Melnik O., Vasilieva E., Kudryavtsev I. (2021). Imbalanced Immune Response of T-Cell and B-Cell Subsets in Patients with Moderate and Severe COVID-19. Viruses.

[103] Kalfaoglu B., Almeida-Santos J., Tye C.A., Satou Y., Ono M. (2020). T-Cell Hyperactivation and Paralysis in Severe COVID-19 Infection Revealed by Single-Cell Analysis. Front. Immunol..

[104] Kudryavtsev I.V., Arsentieva N.A., Batsunov O.K., Korobova Z.R., Khamitova I.V., Isakov D.V., Kuznetsova R.N., Rubinstein A.A., Stanevich O.V., Lebedeva A.A. (2021). Alterations in B Cell and Follicular T-Helper Cell Subsets in Patients with Acute COVID-19 and COVID-19 Convalescents. Curr. Issues Mol. Biol..

[105] Zhang R., Miao J., Zhang K., Zhang B., Luo X., Sun H., Zheng Z., Zhu P. (2022). Th1-Like Treg Cells Are Increased But Deficient in Function in Rheumatoid Arthritis. Front. Immunol..

[106] Gong F., Dai Y., Zheng T., Cheng L., Zhao D., Wang H., Liu M., Pei H., Jin T., Yu D. (2020). Peripheral CD4+ T cell subsets and antibody response in COVID-19 convalescent individuals. J. Clin. Investig..

[107] Melenotte C., Silvin A., Goubet A.-G., Lahmar I., Dubuisson A., Zumla A., Raoult D., Merad M., Gachot B., Hénon C. (2020). Immune responses during COVID-19 infection. Oncoimmunology.

[108] Grant C.R., Liberal R., Mieli-Vergani G., Vergani D., Longhi M.S. (2015). Regulatory T-cells in autoimmune diseases: Challenges, controversies and—yet—unanswered questions. Autoimmun. Rev..

[109] Qiu R., Zhou L., Ma Y., Zhou L., Liang T., Shi L., Long J., Yuan D. (2020). Regulatory T Cell Plasticity and Stability and Autoimmune Diseases. Clin. Rev. Allergy Immunol..

[110] Shah B., Kunal S., Bansal A., Jain J., Poundrik S., Shetty M.K., Batra V., Chaturvedi V., Yusuf J., Mukhopadhyay S. (2022). Heart rate variability as a marker of cardiovascular dysautonomia in post-COVID-19 syndrome using artificial intelligence. Indian Pacing Electrophysiol. J..

[111] Joller N., Lozano E., Burkett P.R., Patel B., Xiao S., Zhu C., Xia J., Tan T.G., Sefik E., Yajnik V. (2014). Treg cells expressing the coinhibitory molecule TIGIT selectively inhibit proinflammatory Th1 and Th17 cell responses. Immunity.

[112] Galván-Peña S., Leon J., Chowdhary K., Michelson D.A., Vijaykumar B., Yang L., Magnuson A.M., Chen F., Manickas-Hill Z., Piechocka-Trocha A. (2021). Profound Treg perturbations correlate with COVID-19 severity. Proc. Natl. Acad. Sci. USA.

[113] van der Ploeg T., Austin P.C., Steyerberg E.W. (2014). Modern modelling techniques are data hungry: A simulation study for predicting dichotomous endpoints. BMC Med. Res. Methodol..

[114] Haunhorst S., Bloch W., Javelle F., Krüger K., Baumgart S., Drube S., Lemhöfer C., Reuken P., Stallmach A., Müller M. (2022). A scoping review of regulatory T cell dynamics in convalescent COVID-19 patients—Indications for their potential involvement in the development of Long COVID?. Front. Immunol..

[115] Wang L., Foer D., MacPhaul E., Lo Y.C., Bates D.W., Zhou L. (2022). PASCLex: A comprehensive post-acute sequelae of COVID-19 (PASC) symptom lexicon derived from electronic health record clinical notes. J. Biomed. Inform..

[116] Aquino A., Zaikova E., Kalinina O., Karonova T.L., Rubinstein A., Mikhaylova A.A., Kudryavtsev I., Golovkin A.S. (2024). T Regulatory Cell Subsets Do Not Restore for One Year After Acute COVID-19. Int. J. Mol. Sci..

[117] Hoffmann A.D., Weinberg S.E., Swaminathan S., Chaudhuri S., Almubarak H.F., Schipma M.J., Mao C., Wang X., El-Shennawy L., Dashzeveg N.K. (2023). Unique molecular signatures sustained in circulating monocytes and regulatory T cells in convalescent COVID-19 patients. Clin. Immunol..

[118] Ryabkova V.A., Churilov L.P., Shoenfeld Y. (2021). Influenza infection, SARS, MERS and COVID-19: Cytokine storm—The common denominator and the lessons to be learned. Clin. Immunol..

[119] Scarpino I., Zucco C., Vallelunga R., Luzza F., Cannataro M. (2022). Investigating topic modeling techniques to extract meaningful insights in Italian long COVID narration. BioTech.

[120] Dovgalova A., Stroev Y.I., Churilov L.P. (2022). Subacute de Quervain’s thyroiditis and COVID-19. Health Is Basis Hum. Potential Probl. Solut..

[121] Meng Q.T., Song W.Q., Churilov L.P., Zhang F.M., Wang Y.F. (2023). Psychophysical therapy and underlying neuroendocrine mechanisms for the rehabilitation of long COVID-19. Front. Endocrinol..

[122] Sjaastad L.E., Owen D.L., Tracy S.I., Farrar M.A. (2021). Phenotypic and Functional Diversity in Regulatory T Cells. Front. Cell Dev. Biol..

[123] Sengupta S., Loomba J., Sharma S., Brown D.E., Thorpe L., A Haendel M., Chute C.G., Hong S. (2022). Analyzing historical diagnosis code data from NIH N3C and RECOVER programs using deep learning to determine risk factors for Long COVID. 2022 IEEE International Conference on Bioinformatics and Biomedicine (BIBM).

[124] Ahmad T., Chaudhuri R., Joshi M.C., Almatroudi A., Rahmani A.H., Ali S.M. (2020). COVID-19: The Emerging Immunopathological Determinants for Recovery or Death. Front. Microbiol..

[125] Jha M., Gupta R., Saxena R. (2023). A precise method to detect Post-COVID-19 pulmonary fibrosis through extreme gradient boosting. SN Comput. Sci..

[126] Banda J.M., Adderley N., Ahmed W.U., AlGhoul H., Alser O., Alser M., Areia C., Cogenur M., Fišter K., Gombar S. (2021). Characterization of long-term patient-reported symptoms of COVID-19: An analysis of social media data. medRxiv.

[127] Pfaff E.R., Girvin A.T., Bennett T.D., Bhatia A., Brooks I.M., Deer R.R., Dekermanjian J.P., Jolley S.E., Kahn M.G., Kostka K. (2022). Identifying who has long COVID in the USA: A machine learning approach using N3C data. Lancet Digit. Health.

[128] Gupta A., Jain V., Singh A. (2022). Stacking Ensemble-Based Intelligent Machine Learning Model for Predicting Post-COVID-19 Complications. New Gener. Comput..

[129] Jiang W., Johnson D., Adekunle R., Heather H., Xu W., Cong X., Wu X., Fan H., Andersson L.M., Robertson J. (2023). COVID-19 is associated with bystander polyclonal autoreactive B cell activation as reflected by a broad autoantibody production, but none is linked to disease severity. J. Med. Virol..

[130] Hill E., Mehta H., Sharma S., Mane K., Xie C., Cathey E., Loomba J., Russell S., Spratt H., DeWitt P.E. (2022). Risk factors associated with post-acute sequelae of SARS-CoV-2 in an EHR cohort: A national COVID cohort collaborative (N3C) analysis as part of the NIH RECOVER program. medRxiv.

[131] Subramanian R., Rubi R.D., Dedeepya M., Gorugantu S.K.V. (2022). Quantitative progression analysis of Post-Acute sequelae of COVID-19, pulmonary fibrosis (PASC-PF) and artificial intelligence driven CT scoring of lung involvement in COVID-19 infection using HRCT-Chest images. Med. Res. Arch..

[132] Sudre C.H., Murray B., Varsavsky T., Graham M.S., Penfold R.S., Bowyer R.C., Pujol J.C., Klaser K., Antonelli M., Canas L.S. (2021). Attributes and predictors of long COVID. Nat. Med..

[133] Stroev Y.U.I., Kaminova O.M., Serdyuk I.Y.U., Nitsa N.A., Chesnokov O.D., Churilov L.P. (2012). Immuno-endokrinnye vzaimodejstviya pri ostryh i hronicheskih zabolevaniyah kak proyavlenie tipovogo konflikta sistemnoj i mestnoj regulyacii [Immune-Endocrine Interactions in Acute and Chronic Diseases as a Manifestation of Typical Conflict Between Systemic and Local Regulation]. Tavricheskiy Med.-Biol. Vesn..

[134] Lu S.Y., Liu K.Y., Liu D.H., Xu L.P., Huang X.J. (2011). High frequencies of CD62L^+^ naive regulatory T cells in allografts are associated with a low risk of acute graft-versus-host disease following unmanipulated allogeneic haematopoietic stem cell transplantation. Clin. Exp. Immunol..

[135] Binka M., Klaver B., Cua G., Wong A.W., Fibke C., García H.A.V., Adu P., Levin A., Mishra S., Sander B. (2022). An Elastic Net Regression Model for Identifying Long COVID Patients Using Health Administrative Data: A Population-Based Study. Open Forum Infect. Dis..

[136] Moreno-Pérez O., Merino E., Leon-Ramirez J.-M., Andres M., Ramos J.M., Arenas-Jiménez J., Asensio S., Sanchez R., Ruiz-Torregrosa P., Galan I. (2021). Post-acute COVID-19 syndrome. Incidence and risk factors: A Mediterranean cohort study. J. Infect..

[137] Miao L., Last M., Litvak M. (2022). An interactive analysis of user-reported long COVID symptoms using Twitter data. Proceedings of the 2nd Workshop on Deriving Insights from User-Generated Text.

[138] Zhang H., Zang C., Xu Z., Zhang Y., Xu J., Bian J., Morozyuk D., Khullar D., Zhang Y., Nordvig A.S. (2023). Data-driven identification of post-acute SARS-CoV-2 infection subphenotypes. Nat. Med..

[139] McCorkell L., Assaf G.S., Davis H.E., Wei H., Akrami A. (2021). Patient-Led research collaborative: Embedding patients in the Long COVID narrative. PAIN Rep..

[140] Wang W., Su B., Pang L., Qiao L., Feng Y., Ouyang Y., Guo X., Shi H., Wei F., Su X. (2020). High-dimensional immune profiling by mass cytometry revealed immunosuppression and dysfunction of immunity in COVID-19 patients. Cell. Mol. Immunol..

[141] Déguilhem A., Malaab J., Talmatkadi M., Renner S., Foulquié P., Fagherazzi G., Loussikian P., Marty T., Mebarki A., Texier N. (2022). Identifying profiles and symptoms of patients with long COVID in France: Data mining infodemiology study based on social media. JMIR Infodemiology.

[142] Suri J.S., Agarwal S., Gupta S.K., Puvvula A., Biswas M., Saba L., Bit A., Tandel G.S., Agarwal M., Patrick A. (2021). A narrative review on characterization of acute respiratory distress syndrome in COVID-19-infected lungs using artificial intelligence. Comput. Biol. Med..

[143] Vinuesa C.G., Linterman M.A., Yu D., MacLennan I.C. (2016). Follicular Helper T Cells. Annu. Rev. Immunol..

[144] Riley R.D., Ensor J., Snell K.I.E., Harrell F.E., Martin G.P., Reitsma J.B., Moons K.G.M., Collins G., Van Smeden M. (2020). Calculating the sample size required for developing a clinical prediction model. BMJ.

[145] Saha P., Talwar P. (2024). Idiopathic pulmonary fibrosis (IPF): Disease pathophysiology, targets, and potential therapeutic interventions. Mol. Cell. Biochem..

[146] Kudlay D.A. (2025). Artificial Intelligence in Pharmaceutical Development.

[147] Karev V., Starshinova A.Y., Glushkova A., Kudlay D., Starshinova A. (2023). Features of myocarditis: Morphological differential diagnosis in post-COVID-19 children. Diagnostics.

